# Accuracy of ultrasonography and magnetic resonance imaging for detection of full thickness rotator cuff tears

**DOI:** 10.4103/0973-6042.63218

**Published:** 2009

**Authors:** Gohar Abbas Naqvi, Mutaz Jadaan, Paul Harrington

**Affiliations:** Our Lady's Hospital, Navan, Ireland

**Keywords:** Magnetic resonance imaging, rotator cuff tear, ultrasonography

## Abstract

**Background:**

Rotator cuff problems are frequently seen by orthopedic surgeons and accurate diagnosis is essential for appropriate management. Value of the clinical assessment of a shoulder is often limited, therefore, imaging studies have important implications in the management of rotator cuff pathologies.

**Aim:**

The purpose of this retrospective study is to compare the accuracy of ultrasonography (US) and magnetic resonance imaging (MRI) for detection of full-thickness rotator cuff tears.

**Materials and Methods:**

We reviewed 91 consecutive cases of shoulder arthroscopy and open rotator cuff repair, who had undergone preoperative investigation in the form of either an ultrasound or MRI. Thirty-six patients had an ultrasound and 55 had an MRI for their affected shoulders. We compared the accuracy of US and MRI for detection of full-thickness rotator cuff tears, using the operative findings as the ‘gold standard’. Data regarding a supraspinatus tear was assessed for the purpose of this study.

**Results:**

Ultrasonography correctly diagnosed 15 out of 17 tears (sensitivity of 0.88). There were 17 true-negative and two false-positive ultrasounds (specificity of 0.89). MRI accurately identified 33 of the 36 tears (sensitivity of 0.91). There were 16 true-negative and three false-positive tears on MRI (specificity of 0.84). The positive predictive value (PPV) was 88% for US and 92% for MRI. The negative predictive value (NPV) was 89% for US and 84% for MRI. The overall accuracy of the ultrasound was 88.89% (95% confidence interval (CI) = 74.09 to 96.18) as compared to 89.09% (95% CI = 77.82% to 95.26%) for the MRI.

**Conclusion:**

Full-thickness rotator cuff tears can be identified using ultrasound and MRI with comparable accuracy. US being a dynamic study and better tolerated by the patient, can therefore be used as the first-line investigation for rotator cuff tear, where appropriate skills are available to reduce the waiting time and cost of investigation.

## INTRODUCTION

Rotator cuff pathologies are frequently encountered in patients presenting with a painful shoulder. The prevalence of shoulder problems in patients presenting to a primary care facility in the United Kingdom, is estimated to be 2.4%.[1] Thirty to seventy percent of such shoulder pain is due to disorders of the rotator cuff.[2,3] This shows how much of a financial burden they present to the healthcare system.

The location of shoulder pain is a poor indicator of its origin, and the value of clinical examination alone is often limited with regard to making a decision for further management with certainty.[4] The results of the imaging of the shoulder may have clinical consequences as the decision to proceed with surgery or to continue conservative management depends on the accurate diagnosis of the extent of the rotator cuff tear. Patients with a partial-thickness tear can be managed with conservative treatment, while patients with a full-thickness tear, with associated weakness of active shoulder abduction, require surgical repair.[4]

The rotator cuff can be visualized with non-invasive imaging techniques such as ultrasonography (US)[5,6] and magnetic resonance imaging (MRI).[6] Initial US results in the detection of rotator cuff tears varied,[7,8] probably due to the use of low frequency transducers and limited experience with the examination procedure. Magnetic resonance imaging quickly became the favored investigation for preoperative diagnosis of partial- and full-thickness rotator cuff tears, with high sensitivity and accuracy.[9,10] Thus, MRI has been considered the imaging modality of choice for evaluating the rotator cuff tears despite its relatively high cost and occasional limited availability.

Subsequently, technical improvements such as 7.5 – 14 MHz linear array broad-bandwidth transducers and better penetration of the ultrasound beam, as well as, increased experience, significantly improved ultrasonographic results and reliability.[11,12]

The purpose of the present study is to compare the accuracy of ultrasound and magnetic resonance imaging, in our institution, for the detection of full-thickness rotator cuff tears (RCTs), using the operative findings as a ‘gold standard’.

## MATERIALS AND METHODS

This retrospective study comprised of 91 consecutive patients with shoulder pain, who had undergone preoperative imaging in the form of US or MRI and subsequently proceeded to arthroscopic or open-shoulder surgery. Thirty six patients had US (21 males and 15 females, mean age 54.33 years), while 55 had MRI (35 males and 20 females, mean age 56.56 years) for their affected shoulder. All the patients subsequently underwent shoulder arthroscopy or open surgery according to the standardized technique, by the senior author. The presence or absence of a full-thickness or partial-thickness supraspinatus tear was documented in the operative notes along with the size of the full-thickness tears. For the purpose of comparison, we divided the operative findings into five categories. These were, intact cuff, a partial-thickness tear, a small full-thickness tear (< 1 cm) grade I, a moderate-full thickness tear (1 – 3 cm) grade II, and a large / massive tear (> 3 cm) grade III.

### Ultrasonography

**Figure 1:**
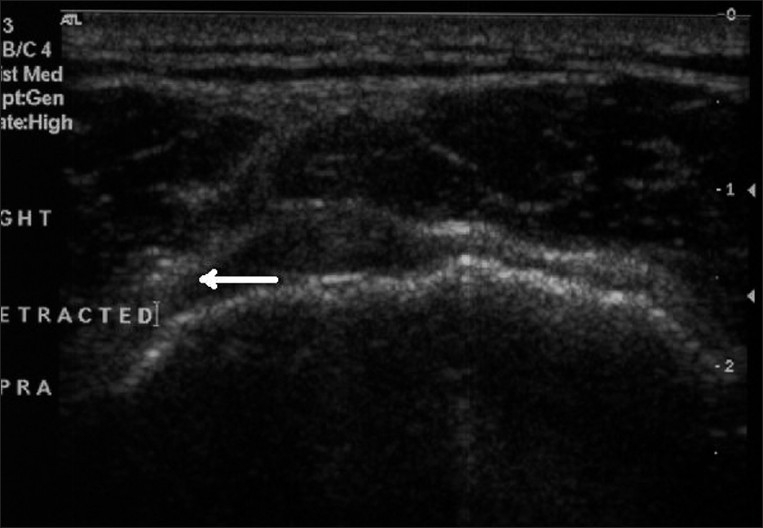
Ultrasound showing full-thickness retracted tear in the right supraspinatus tendon (arrow)

**Figure 2:**
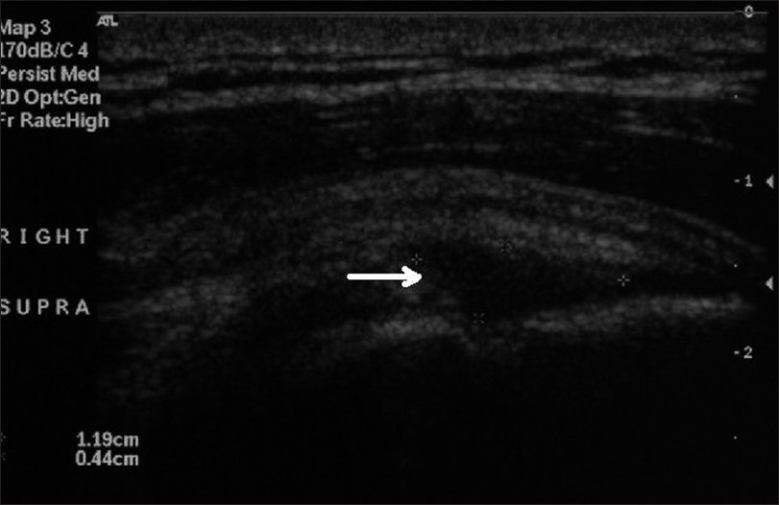
Ultrasound showing partial-thickness rotator cuff tear (arrow), indicated by a distinct hypoechoic defect at the articular surface of the supraspinatus tendon

All ultrasonograms were performed by radiologists experienced in musculoskeletal ultrasound using a high-frequency, linear-array transducer. The finding of a full-thickness rotator cuff tear was recorded when the rotator cuff could not be visualized because of complete avulsion or when there was a focal defect extending from the bursal to the humeral side of the rotator cuff [Figure 1]. A partial-thickness tear was diagnosed when there was flattening of the bursal side of the rotator cuff or a distinct hypoechoic defect visualized at the articular side of the rotator cuff [Figure 2].

### Magnetic resonance imaging

**Figure 3:**
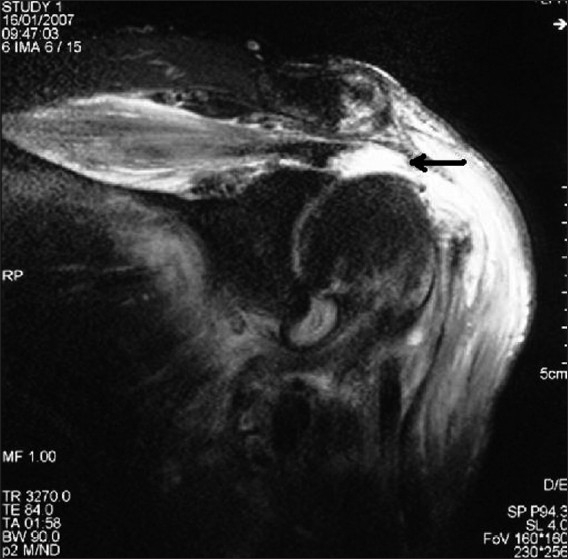
Coronal-oblique T2-weighted magnetic resonance image showing full-thickness rotator cuff tear (arrow), indicated by high signal intensity fluid replacing the insertion of the supraspinatus tendon at the greater tuberosity

Multi-planar MR imaging of the shoulder was performed using coronal oblique proton density, coronal oblique T1 weighted, coronal oblique T2 weighted with fat saturation, sagittal oblique T2-weighted with fat saturation, and axial T2 weighted sequences. All MRIs were reported by a radiologist with special interest in musculoskeletal imaging. The criterion for a full-thickness rotator cuff tear was a focal, well-defined area of increased signal intensity on T1-weighted and T2-weighted images that extended from the bursal to the articular surface [Figure 3]. A partial-thickness tear was defined when the fluid signals donot traverse the full thickness of the tendon.

### Data analysis

All rotator cuff tendons were assessed during imaging, but only the integrity of the supraspinatus tendon, which was the most commonly involved tendon, was analyzed for the purpose of this study. The results of US and MRI were considered as negative when there was no tear or a partial-thickness tear and positive when a full-thickness tear was found, for the purpose of this study. The results of US and MRI were compared separately with the operative findings and were scored in a similar manner.

The accuracy of US and MRI (percentage of correct diagnosis) was calculated with 95% confidence intervals (95% CI). The sensitivity, specificity, and positive and negative predictive values (PPV and NPV) were also calculated for the diagnosis of full-thickness rotator cuff tears. Statistical analysis was not performed separately for partial-thickness tears, due to the limited number in this study.

## RESULTS

Ultrasonography diagnosed 15 out of 17 tears (sensitivity of 0.88) accurately. There were 17 true-negative and two false-positive ultrasounds (specificity of 0.89) [Tables 1 and 2]. The MRIs accurately identified 33 of the 36 tears (sensitivity of 0.91). There were 16 true-negative and three false-positive tears on MRI (specificity of 0.84) [Tables 1 and 3]. The positive predictive value (PPV) was 88% for US and 92% for MRI. The negative predictive value (NPV) was 89% for US and 84% for MRI. The overall accuracy of the ultrasound was 88.89% (95% confidence interval (CI) = 74.09 to 96.18) as compared to 89.09% (95% CI = 77.82% to 95.26%) for MRI.

**Table 1:** Comparision of ultrasonography and magnetic resonance imaging with operative findings for rotator cuff tear

Operative findings	Ultrasound				MRI			
	
	Full-thickness tear	Partial-thickness tear	No tear	Total	Full-thickness tear	Partial-thickness tear	No tear	Total
Full-thickness tears	15	0	2	17	33	1	2	36
Partial-thickness tears	0	4	0	4	1	3	1	5
No tear	2	2	11	15	2	0	12	14
Total	17	6	13	36	36	4	15	55

**Table 2:** Accuracy of ultrasonography for detection of full-thickness rotator cuff tears

Operative findings	Ultrasonographic findings		

	Full-thickness tear	Partial-thickness and no tear	Total
Full-thickness tears	15	2	17
Partial-thickness and no tear	2	17	19
Total	17	6	36

**Table 3:** Accuracy of MRI for detection of full-thickness rotator cuff tears

Operative findings	MRI findings		

	Full-thickness tear	Partial-thickness and no tear	Total
Full-thickness tears	33	3	36
Partial-thickness and no tear	3	16	19
Total	36	19	55

## DISCUSSION

Rotator cuff pathologies are frequently encountered in patients presenting with shoulder pain, and account for 30 to 70% of these cases.[2,3] When assessing a patient with shoulder pain and sign of impingement, it is important to ascertain the integrity of the rotator cuff and the extent of the tear, if there is one. This information allows the surgeons to plan a strategy for further management of the patient. Traditionally, arthrography has been used for the diagnosis of rotator cuff tears.[13,14] However, it is an invasive technique with well-recognized risks and has largely been replaced by US and MRI. Both US and MRI have shown comparable results in detecting RCTs, demonstrating an accuracy of 87% and sensitivities and specificities of over 90%.[15–18]

Ultrasonography of the shoulder was first reported by Seltzer *et al*.[19] Since then several authors have been discussing and refining this method.[20,21] There are several advantages of US over MRI. Ultrasonography has the benefit of being a dynamic form of imaging as compared to the static MRI. US is portable, quick, and a more cost-effective method, which is also better tolerated by the patient and allows interaction with the patient, who can point at the symptomatic area, which will optimize the diagnostic yield. The advent of portable US scanners has made it possible for orthopedic surgeons to acquire the skill and perform US in the clinic at the first point of contact. This saves an enormous amount of time and money and reduces the work load and financial burden on the Radiology Department. Al-shawi *et al*.[22] have studied 143 consecutive patients with shoulder problems, who underwent shoulder US by an orthopedic surgeon, and reported a sensitivity and specificity of 96.2% and 95.4%, respectively. Joseph *et al*.[23] reported 88% accuracy for full-thickness and 70% for partial-thickness tears, with the use of office-based ultrasonography by an orthopedic surgeon.

The present study was designed to evaluate the accuracy of US and MRI for rotator cuff tears, with the use of updated imaging technology. The limitation of this study is that, being a retrospective study, the patient would have undergone either US or MRI as part of their preoperative assessment and not both.

In the US group, (N = 36) 17 had full thickness RCT, 15 were diagnosed accurately, while two were diagnosed with no tear. All four partial-thickness tears were accurately identified by US. Out of 15 intact cuffs 11 were diagnosed accurately, two were diagnosed as partial-thickness tears, and two as full-thickness tears. In the MRI group, (N = 55) 33 of the 36 full-thickness tears were accurately diagnosed. Of the five partial-thickness tears, three were correctly diagnosed, one was diagnosed as a full-thickness tear, and one as intact tendon.

Both imaging modalities showed comparable accuracy for detecting full-thickness RCTs. Due to the limited number of partial-thickness tears in this study, a statistical analysis could not be performed separately, and they were considered as ‘no tear,’ for statistical analysis.

## CONCLUSION

Full-thickness rotator cuff tears can be identified using ultrasound and MRI with comparable accuracy. US being less expensive, less time-consuming, more dynamic, and less demanding for patients, should be used as the first-line of investigation for rotator cuff tear, where appropriate skills are available.

## References

[1] Linsell L, Dawson J, Zondervan K, Rose P, Randall T, Fitzpatrick R (2006). Prevalence and incidence of adults consulting for shoulder conditions in UK primary care: Patterns of diagnosis and referral. Rheumatology (Oxford).

[2] Mitchell C, Adebajo A, Hay E, Carr A (2005). Shoulder pain: Diagnosis and management in primary care. BMJ.

[3] Macfarlane GJ, Hunt IM, Silman AJ (1998). Predictors of chronic shoulder pain: A population based prospective study. J Rheumatol.

[4] Dalton SE (1994). The conservative management of rotator cuff disorders. Br J Rheumatol.

[5] Crass JR, Craig EV, Bretzke C, Feinberg SB (1985). Ultrasonography of the rotator cuff. Radiographics.

[6] Seibold CJ, Mallisee TA, Erickson SJ, Boynton MD, Raasch WG, Timins ME (1999). Rotator cuff: evaluation with US and MR imaging. Radiographics.

[7] Brandt TD, Cardone BW, Grant TH, Post M, Weiss CA (1998). Rotator cuff sonography: A reassessment. Radiology.

[8] Seltzer SE, Finberg HJ, Weissman BN, Kido DK, Collier BD (1979). Arthrography: Gray-scale ultrasound evaluation of the shoulder. Radiology.

[9] Iannotti JP, Zlatkin MB, Esterhai JL, Kressel HY, Dalinka MK, Spindler KP (1991). Magnetic resonance imaging of the shoulder. J Bone Joint Surg Am.

[10] Singson RD, Hoang T, Dan S, Friedman M (1996). MR evaluation of rotator cuff pathology using T2-weighted fast spin-echo technique with and without fat suppression. AJR Am J Roentgenol.

[11] Teefey SA, Rubin DA, Middleton WD, Hildebolt CF, Leibold RA, Yamaguchi K (2004). Detection and quantification of rotator cuff tears: Comparison of ultrasonographic, magnetic resonance imaging, and arthroscopic findings in 71 consecutive cases. J Bone Joint Surg Am.

[12] Jacobson JA, Lancaster S, Prasad A, van Holsbeeck MT, Craig JG, Kolowich P (2004). Full-thickness and partial-thickness supraspinatus tendon tears: Value of US signs in diagnosis. Radiology.

[13] Mink JH, Harris E, Rappaport M (1985). Rotator cuff tears: Evaluation using double-contrast shoulder arthrography. Radiology.

[14] Goldman AB, Ghelman B (1978). The double-contrast shoulder arthrogram: A review of 158 studies. Radiology.

[15] Teefey SA, Rubin DA, Middleton WD, Hildebolt CF, Leibold RA, Yamaguchi K (2004). Detection and quantification of rotator cuff tears. J Bone Joint Surg Am.

[16] Swen WA, Jacobs JW, Algra PR, Manoliu RA, Rijkmans J, Willems WJ (1999). Sonography and magnetic resonance imaging equivalent for the assessment of full-thickness rotator cuff tears. Arthritis Rheum.

[17] Teefey SA, Hasan SA, Middleton WD, Patel M, Wright RW, Yamaguchi K (2000). Ultrasonography of the rotator cuff: A comparison of ultrasonographic and arthroscopic findings in one hundred consecutive cases. J Bone Joint Surg Am.

[18] Bachmann GF, Melzer C, Heinrichs CM, Möhring B, Rominger MB (1997). Diagnosis of rotator cuff lesions: Comparison of US and MRI on 38 joint specimens. Eur Radiol.

[19] Seltzer SE, Finberg HJ, Weissman BN (1980). Arthrosonography: Technique, sonographic anatomy and pathology. Invest Radiol.

[20] Crass JR, Craig EV, Bretzke C, Feinberg SB (1985). Ultrasonography of the rotator cuff. Radiographics.

[21] Middleton WD, Reinus WR, Totty WG, Melson CL, Murphy WA (1986). Ultrasonographic evaluation of the rotator cuff and biceps tendon. Bone Joint Surg Am.

[22] Al-Shawi A, Badge R, Bunker T (2008). The detection of full thickness rotator cuff tear using ultrasound. J Bone Joint Surg Br.

[23] Iannotti JP, Ciccone J, Buss DD, Visotsky JL, Mascha E, Cotman K (2005). Accuracy of office-based ultrasonography of the shoulder for the diagnosis of rotator cuff tears. J Bone Joint Surg Am.

